# Exit from Arsenite-Induced Mitotic Arrest Is p53 Dependent

**DOI:** 10.1289/ehp.8969

**Published:** 2006-06-23

**Authors:** Samuel C. McNeely, Xiaogiang Xu, B. Frazier Taylor, Wolfgang Zacharias, Michael J. McCabe, J. Christopher States

**Affiliations:** 1 Department of Pharmacology and Toxicology; 2 Department of Medicine; 3 James Graham Brown Cancer Center and; 4 Center for Genetics and Molecular Medicine, University of Louisville, Louisville, Kentucky, USA; 5 Department of Environmental Medicine, University of Rochester, Rochester, New York, USA

**Keywords:** apoptosis, arsenite, ID1, microarray, mitotic arrest, p53

## Abstract

**Background:**

Arsenic is both a human carcinogen and a chemotherapeutic agent, but the mechanism of neither arsenic-induced carcinogenesis nor tumor selective cytotoxicity is clear. Using a model cell line in which p53 expression is regulated exogenously in a tetracycline-off system (TR9-7 cells), our laboratory has shown that arsenite disrupts mitosis and that p53-deficient cells [p53^(−)^], in contrast to p53-expressing cells [p53^(+)^], display greater sensitivity to arsenite-induced mitotic arrest and apoptosis.

**Objective:**

Our goal was to examine the role p53 plays in protecting cells from arsenite-induced mitotic arrest.

**Methods:**

p53^(+)^ and p53^(−)^ cells were synchronized in G_2_ phase using Hoechst 33342 and released from synchrony in the presence or absence of 5 μM sodium arsenite.

**Results:**

Mitotic index analysis demonstrated that arsenite treatment delayed exit from G_2_ in p53^(+)^ and p53^(−)^ cells. Arsenite-treated p53^(+)^ cells exited mitosis normally, whereas p53^(−)^ cells exited mitosis with delayed kinetics. Microarray analysis performed on mRNAs of cells exposed to arsenite for 0 and 3 hr after release from G_2_ phase synchrony showed that arsenite induced inhibitor of DNA binding-1 (ID1) differentially in p53^(+)^and p53^(−)^ cells. Immunoblotting con-firmed that ID1 induction was more extensive and sustained in p53^(+)^ cells.

**Conclusions:**

p53 promotes mitotic exit and leads to more extensive ID1 induction by arsenite. ID1 is a dominant negative inhibitor of transcription that represses cell cycle regulatory genes and is elevated in many tumors. ID1 may play a role in the survival of arsenite-treated p53^(+)^ cells and contribute to arsenic carcinogenicity.

Arsenic is both a human carcinogen causing cancer in multiple tissues [National Research Council (NRC) 1999, 2001] and a chemo-therapeutic agent used in the treatment of acute promyelocytic leukemia (Cohen et al. 2001). Arsenic trioxide shows promise for chemotherapy in treatment of solid tumors and inhibits tumor growth in an orthotopic prostate cancer model (Maeda et al. 2001). However, the mechanism by which arsenic selectively induces cell death in tumor cells is not understood.

Clinically achievable concentrations of arsenite induce cell death in a variety of cancer cell lines and virally immortalized cell lines that lack normal cell cycle control. Arsenic trioxide (As_2_O_3_) induces apoptosis in several prostate and ovarian cancer cell lines (Du and Ho 2001; Uslu et al. 2000). Arsenite induces mitotic arrest and apoptosis in SV40-transformed human fibroblasts (States et al. 2002), spontaneously immortalized p53-deficient Li-Fraumeni human fibroblasts (Taylor et al. 2006), and in HeLa S3 and KB cells (Huang and Lee 1998). In contrast, these moderate concentrations of arsenite slow the growth of diploid human fibroblasts but do not induce cell death (States et al. 2002; Yih et al. 1997). However, arsenite disrupts normal mitotic progression and induces aneuploidy in human diploid fibroblasts (Yih et al. 1997) and peripheral blood lymphocytes (Vega et al. 1995).

A common feature of the cell lines in which arsenite induces apoptosis is a p53-deficient phenotype, either by mutation or viral oncogene inactivation of p53. p53 regulates cell cycle progression and apoptosis in response to genetic damage. The molecular mechanisms by which p53 arrests cell cycle progression in G_1_ and G_2_ phases in response to DNA damage are reasonably well understood (Jin and Levine 2001). p53 is activated in response to mitotic disruption by arsenic (Yih and Lee 2000). However, the signals for p53 activation and the role that p53 plays in the cellular response to mitotic disruption are not clear.

In asynchronous TR9-7 cells (human fibroblasts with tetracycline-regulated p53 expression), arsenite treatment induces a p53-independent accumulation of cells with G_2_/M phase DNA content (Taylor et al. 2006). However, accumulation of mitotic cells occurs in p53-deficient [p53^(−)^] but not in p53-expressing [p53^( + )^] TR9-7 cells. Furthermore, the mitotically arrested p53^(−)^ cells undergo apoptosis, whereas most p53^(+)^ cells exit G_2_/M phase and arrest in G_1_ phase. Therefore, we hypothesized that p53 did not prevent delayed G_2_ transit but did protect against apoptosis subsequent to the arsenite-induced mitotic arrest.

To investigate the role of p53 in the arsenite-induced disruption of G_2_/M phase progression, we examined the response of p53^(+)^ and p53^(−)^ TR9-7 cells to arsenite treatment after release from G_2_ phase synchrony. We show that in synchronized TR9-7 cells arsenite delayed entry into mitosis independently of p53 expression but that the exit from mitosis in arsenite-treated cells was p53 dependent. Furthermore, we show that the apoptotic process occurred while the cells attempted cytokinesis. We hypothesized the difference in exit kinetics and resistance to apoptosis between p53^(+)^ and p53^(−)^ cells was due to p53-mediated alterations of gene expression profiles. Microarray analysis of G_2_ phase–synchronized p53^(+)^ and p53^(−)^ cells treated with arsenite for 3 hr revealed p53-dependent induction of inhibitor of DNA binding-1 (ID1). Negative transcriptional regulation of cyclin-dependent kinase inhibitors by ID1 may have implications for cell cycle progression in p53^(+)^ cells surviving arsenite-induced mitotic perturbation and may play a role in arsenic-induced carcinogenesis.

## Materials and Methods

### Cell culture and synchronization

TR9-7 cells were the gift of M.A. Tainsky (Wayne State University, Detroit, MI). Cells were cultured at 37ºC at 5% CO_2_ in a humidified incubator in alpha modification of minimal essential media (GIBCO/BRL, Gaithersburg, MD) supplemented with 10% fetal bovine serum (HyClone, Logan, UT), 10 mM HEPES (pH 7.0), 200 μg/mL geneticin (Invitrogen Corp., Carlsbad, CA), 40 μg/mL hygromycin B (Sigma Chemical Co., St. Louis, MO), 100 U/mL penicillin, and 10 U/mL streptomycin.

Cells were synchronized in G_2_ phase of the cell cycle according to adaptation of published procedures (Kuhholzer and Prather 2001; Tobey et al. 1990). Briefly, we plated cells in the presence of 15 ng/mL tetracycline (Fisher Scientific, Pittsburgh, PA) to allow moderate p53 expression. We replaced media with media containing 5 μg/mL aphidicolin (Sigma) and incubated the cells for 24 hr. We then washed the cells with phosphate-buffered saline (PBS) and incubated them in media containing 0.1 μg/mL Hoechst 33342 (Sigma) for 12 hr. After 6 hr of incubation with Hoechst 33342, p53 expression was suppressed in half the cultures by direct addition of tetracycline (1 mg/mL) to the media to a final concentration of 1,500 ng/mL. Cells were released from G_2_ phase by washing with PBS and refeeding with fresh drug-free media.

### Arsenic compounds

We prepared fresh working aqueous solutions of sodium arsenite (NaAsO_2_) (Sigma) on the day of treatment and filter sterilized prior to use.

### Flow cytometry

For DNA content analysis, cells were harvested by trypsinization and fixed in 1 mL ice-cold 70% ethanol. Following fixation overnight at 4ºC, we centrifuged the samples at 1,500 × *g* and resuspended the resulting cell pellets in PBS containing propidium iodide and 100 U/mL RNase A (Sigma) for 30 min at room temperature. We analyzed propidium iodide–stained samples on a FACSCalibur (Becton Dickinson and Co., San Jose, CA) using doublet discrimination. Propidium iodide fluorescence was collected on channel two (FL2) (585/42 nm) using linear amplification. A minimum of 20,000 events/sample was analyzed. Data were collected using CellQuest software (Becton Dickinson) and analyzed for cell cycle distribution using Modfit software (Verity Software House, Inc., Topsham, ME).

### Mitotic indices

Cells were harvested for mitotic index analysis by trypsinization. Media, wash, and cells were collected together and centrifuged. We resuspended cell pellets in 150 μL serum free media and added 2.5 mL of 0.4% KCl. We incubated the suspension for 10 min at 37ºC, then added methanol:acetic acid fixative solution (3:1, vol/vol) to 2% (vol/vol) and collected cells by centrifugation. Cells were resuspended in 2.5 mL fixative solution and fixed at room temperature for 20 min. Samples were centrifuged; cell pellets were resuspended in 0.5 mL fixative and chilled on ice for 1 hr. Aliquots of the suspensions were dropped onto glass slides, air dried, and stained with Wright Giemsa solution (Fisher). We examined slides under a microscope and counted a minimum of 200 cells to determine mitotic index.

### Western blot analyses

Media from culture dishes were removed and collected along with one PBS wash. Floating cells were pelleted via centrifugation, washed once with PBS, and lysed with lysis buffer [10 mM Tris–HCl (pH 7.4), 1 mM Na_2_–EDTA, 0.1% SDS, 180 μg/mL phenylmethylsulfonyl fluoride]. Adherent cells were lysed directly in the plates. Lysates from floating and adherent fractions were then combined. Protein concentration in the lysates was determined by Bradford assay (BioRad Laboratories, Inc., Hercules, CA). Proteins were resolved by SDS–PAGE in 12% polyacrylamide gels and transferred to supported nitrocellulose membranes by electro-blotting. p53, β-actin, and ID1 were detected by probing membranes with antibodies for p53 (LabVision, Fremont, CA), β-actin (Sigma), or ID1 (Santa Cruz Biotechnologies, Santa Cruz, CA), respectively. Blots were incubated with secondary anti-mouse or anti-rabbit antibody conjugated to horse radish peroxidase (Zymed Laboratories, San Francisco, CA) and visualized with enhanced chemiluminescence (Amersham Biosciences Inc., Piscataway, NJ).

### Statistical analyses

Comparison of mitotic indices of arsenite-treated p53^(+)^ and p53^(−)^ cells was performed by Student *t*-test using SlideWrite software (version 6.10; Advanced Graphics Software, Encinitas, CA) and confirmed by analysis of variance (ANOVA).

### Microarray analyses

We harvested cells via trypsinization and prepared poly(A)^+^ mRNAs using Micro-FastTrack (Invitrogen Corp.). SuperScript II (Invitrogen Corp.) was used with an oligo(dT) primer linked to the T7 RNA polymerase-binding site sequence to synthesize cDNAs. cDNAs were extracted with phenol:chloroforam:isoamyl alcohol (25:24:1), precipitated with NH_4_OAc and ethanol, and resuspended in RNase-free water. We synthesized biotin-labeled cRNA with an ENZO RNA transcript labeling kit (Enzo Life Sciences, Farmingdale, NY), using 0.7 μg cDNA per reaction. cRNAs were purified with RNeasy purification columns (Qiagen, Valencia, CA). cRNA, 15 μg per sample, was heated at 80ºC for 33 min in fragmentation buffer (Affymetrix, Inc., Santa Clara, CA). Fragmented cRNAs were hybridized to U95Av2 GeneChips (Affymetrix) for 16 hr at 45ºC. GeneChips were stained with streptavidin phycoerythrin stain solution (Affymetrix). Signal was amplified with goat anti-streptavidin antibody and biotinylated goat IgG (Affymetrix). We scanned stained GeneChips on an Agilent GeneArray Scanner (Agilent Technologies, Palo Alto, CA) and performed four biological replicates.

We performed ANOVA of the signal data with Bioconductor/R software using MAS (Microarray Suite software) background correction, quantiles normalization, MAS PM (perfect match) correction, MAS summary, and statistical significance defined as *p* < 0.05. Data were filtered on p53 status and arsenite exposure. Analysis was conducted with and without application of the Benjamini and Hochberg false discovery rate correction. Venn analysis was conducted on gene lists using GeneSpring software (Agilent Technologies).

## Results

To determine the role that p53 plays in preventing arsenite-induced accumulation of mitotic cells and in preventing arsenite-induced apoptosis, TR9-7 cells were synchronized in G_2_ using Hoechst 33342. Hoechst 33342 is a topoisomerase inhibitor that interferes with chromatin condensation, resulting in G_2_ phase arrest (Kuhholzer and Prather 2001). The effects of arsenite treatment on the progression from G_2_ phase and through mitosis of synchronized p53^(+)^) and p53^(−)^ cells were evaluated after release from Hoechst 33342-induced G_2_ phase synchronization.

TR9-7 cells are a spontaneously immortalized human fibroblast cell line, derived from a Li-Fraumeni patient, and subsequently stably transfected with a tetracycline-regulated p53 expression vector (Yin et al. 1992). p53 expression in TR9-7 cells is inversely proportional to the tetracycline concentration in the media (tet-off). Absence of tetracycline strongly induces p53 and causes TR9-7 cells to arrest in G_1_ and G_2_ phases (Agarwal et al. 1995). Tetracycline at a concentration of 15 ng/mL induces p53 to a modest level that allows the cells to grow at a rate comparable to p53^(−)^ TR9-7 cells (Taylor et al. 2006).

### Synchronization of TR9-7 cells in G_2_ phase using Hoechst 33342

**Figure 1:**
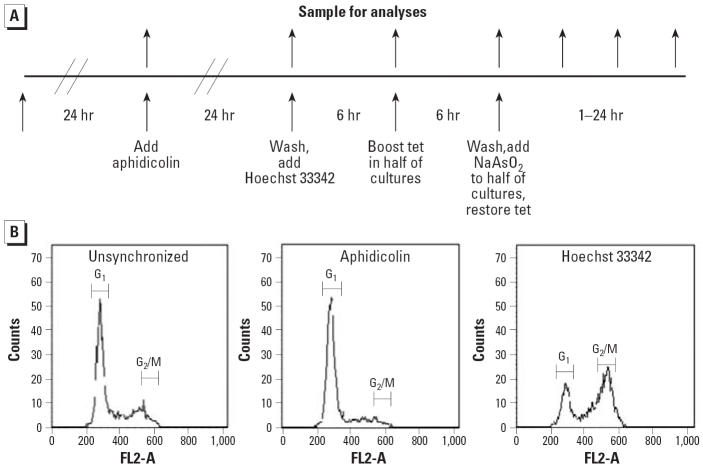
Synchronization of TR9-7 cells expressing or not expressing p53 in G_2_ phase. Abbreviations: FL2-A, propidium iodide fluorescence on channel two; Tet, tetracycline. (*A*) Experimental design. (*B*) Cell cycle distribution of synchronized cells. Samples were harvested after treatments indicated and analyzed by flow cytometry. Positions of cells with G_1_ and G_2_/M phase DNA content are indicated.

**Figure 2:**
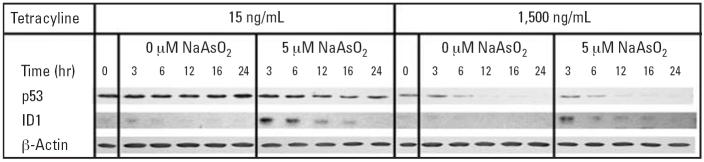
p53 and ID1 expression in cells after G_2_ phase synchronization. Cells were synchronized, treated with 5 μM NaAsO_2_, and samples were taken at the indicated times. Levels of p53 and ID1 proteins were assessed by Western blot analysis with β-actin as a loading control. 0 hr = release from Hoechst 33342. A representative example is shown.

**Figure 3:**
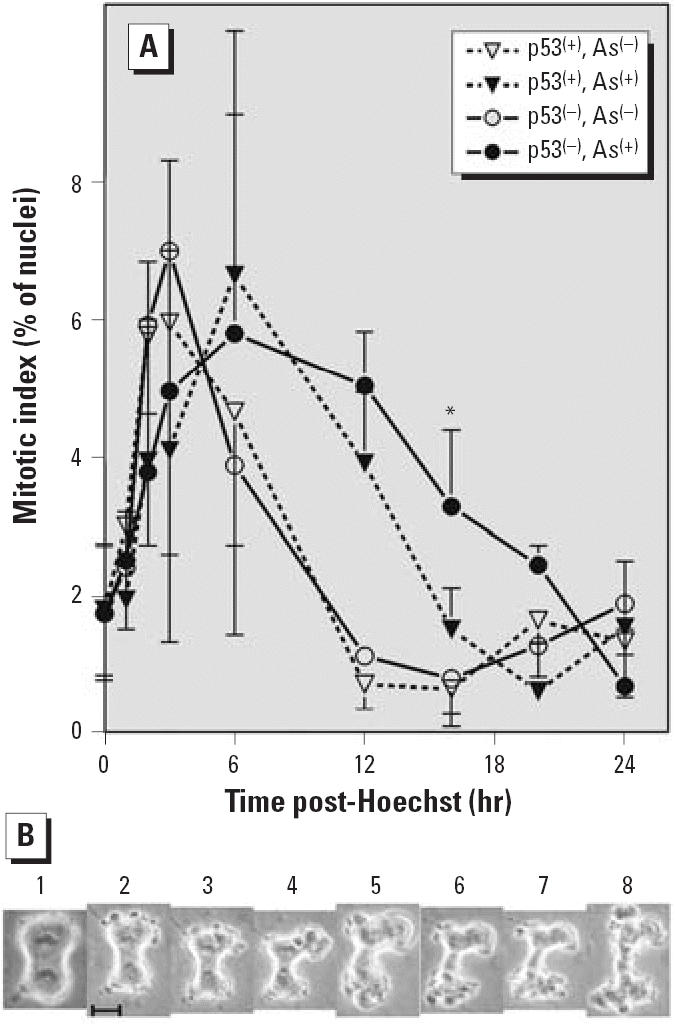
Arsenite treatment delays mitotic entry, and p53 deficiency delays mitotic exit in arsenite-treated cells. Abbreviations: As^(+)^, + 5 μg sodium arsenite; As^(−)^, 0 μg sodium arsenite. (*A*) Mitotic indices of TR9-7 cells released from G_2_ phase synchronization. Mean ± SD from four independent experiments performed with triplicate cultures are plotted. Not all time points assayed in every experiment. (*B*) Arsenite-treated TR9-7 cells in late anaphase/early telophase undergoing apoptosis. Cells were released from G_2_ phase by feeding with fresh media containing 5 μM NaAsO_2_ and monitored by phase microscopy. Photographs in panels 1–8 were taken in order over a 30-min period. Bar, 5 μm. *Significant difference between arsenite-exposed p53(+) and p53(^−^) cells.

Synchronization of TR9-7 cells in G_2_ phase required plating cells with moderate p53 expression. Cells with high levels of p53 tended to arrest in G_1_ or G_2_ phase and not re-enter the cell cycle. In addition, induction of p53 by lowering the tetracycline concentration proved difficult to time reproducibly. We obtained reproducible changes in p53 expression by repressing p53 by addition of tetracycline (data not shown). The experimental design we developed is presented in Figure 1. Cells were synchronized in G_1_ phase by addition of aphidicolin after allowing freshly plated cells to attach and to acclimate for 24 hr. Cells were released from G_1_ phase synchrony after 24-hr aphidicolin exposure by washing the cells and applying fresh media containing Hoechst 33342, which induced G_2_ phase synchrony. After 6 hr of incubation in Hoechst 33342 media, p53 transcription was suppressed in half the cultures by addition of tetracycline to 1,500 ng/mL. Cells were incubated for an additional 6 hr. At this point, media were changed again and half of each tetracycline group received media containing 0 or 5 μM NaAsO_2_ with tetracycline appropriate to maintain p53 expression levels. Samples were taken at each change of media and at several time points post-Hoechst 33342 incubation. Samples were analyzed with flow cytometry to determine cell cycle distribution (Figure 1B), by Western blot to determine p53 and ID1 expression (Figure 2), and for mitotic spread analysis to determine mitotic index (Figure 3A). We performed microarray analysis to determine changes in gene expression (Tables 1–3).

### Cell cycle distribution of synchronized cells

Using the procedure depicted in Figure 1A, we were able to achieve reasonably good synchronization. Unsynchronized TR9-7 cells show a typical cell cycle distribution with a majority of the cells in G_1_ phase and a distinct but small G_2_/M phase peak (Figure 1B). After aphidicolin treatment, > 90% of cells were in the G_1_ phase (Figure 1B). After incubation with Hoechst 33342, approximately 60% of the cells were in G_2_ phase, as characterized using ModFit software to analyze the flow cytometry data (Figure 1B). The approximately 40% of cells remaining in G_1_ phase appeared irreversibly arrested in G_1_.

### p53 expression in cells after G_2_ synchronization

p53 levels were assessed in cells during synchronization and after release from G_2_ phase. Generally, p53 expression was maintained consistently in cultures with low tetra-cycline and suppressed by tetracycline at 1,500 ng/mL throughout the experiment (Figure 2). The achievement of a high level of synchrony in the cultures required the moderate expression of p53 that was suppressed by addition of tetracycline at 1,500 ng/mL 6 hr prior to release from G_2_ phase blockade. A modest amount of residual p53 was detectable in 1,500 ng/mL tetracylcline cultures [p53^(−)^] at 0 and 3 hr after release, although p53 expression was much lower than that observed in cultures with only 15 ng/mL tetracycline [p53^(+)^].

### Arsenite delays mitotic exit selectively in p53^(−)^ cells

We examined arsenite effects on entry into and exit from mitosis in synchronized cells. Mitotic indices and p53 expression were determined in triplicate cultures exposed or not exposed to 5 μM NaAsO_2_ and harvested 0, 1, 3, 6, 12, 16, 20 and 24 hr after release from G_2_ phase synchrony induced by Hoechst 33342 exposure. Mitotic indices were determined only in experiments in which the p53 expression was maintained as expected. The mitotic index of cells not exposed to arsenite peaked 3 hr after release from G_2_ phase (Figure 3A). There was no difference between p53^(+)^ and p53^(−)^ cells. The mitotic index dropped sharply after 3 hr and was < 1% by 12 hr. The mitotic index started to increase again 24 hr after release from G_2_ phase, indicating that the cells had started to cycle again. Arsenite treatment delayed the peak in mitotic index to 6 hr after release from G_2_ phase in both p53(+) and p53^(−)^ cells. The mitotic index of p53^(+)^ cells treated with arsenite decreased after 6 hr and returned to baseline by 16 hr. The slope of the decrease in mitotic index of arsenite-treated p53^(+)^ cells is similar to that of the decline in cells not exposed to arsenite. These results suggest that entry into mitosis is delayed in p53^(+)^ cells but that mitotic exit is normal. In contrast, the mitotic index of p53^(−)^ cells treated with arsenite dropped slowly and was still significantly elevated 16 hr (*p* < 0.001, *n* = four experiments) after release from G_2_ phase arrest. The mitotic index dropped to nearly zero by 24 hr after release from G_2_ phase.

### Arsenite induces alteration of expression profiles

Microarray analysis was conducted on mRNAs prepared from p53^(+)^ and p53^(−)^ cells at 0 and 3 hr after release from G_2_ phase synchrony with or without arsenite exposure. ANOVA of four biological replicates using a *p* < 0.05 was performed with Bioconductor/R software using MAS background correction, quantiles normalization, MAS PM correction, and MAS summary. Analysis was conducted with (Table 1) and without (Tables 2, 3) application of the Benjamini and Hochberg false discovery rate (FDR) correction. Table 1 is all inclusive, whereas Table 2 includes only those genes showing ≥2-fold increase in either p53^(+)^ or p53^(−)^ samples. Arsenite induced several genes independently of p53 status. Heme oxygenase 1 (*HMOX1*) showed a dramatic > 25-fold increase in expression. Metallothioneines 2A and 1X and the zinc transporter *SLC30A1* were also induced. Expression of the stress-responsive heat shock protein *HSP70B* was elevated > 7-fold. ID1 was induced 3.7- to 6.9-fold in p53^(+)^ cells and 2.5- to 4.3-fold in p53^(−)^ cells depending on which of three probe sets was evaluated. Although two-way ANOVA did not indicate that the difference in fold-change between p53^(+)^ and p53^(−)^ cells was statistically significant, Western blot analysis did show ID1 induction that was more extensive and sustained in p53^(+)^ cells (Figure 2). Two-way ANOVA did not identify any genes or which expression was dependent on p53; therefore, Venn analysis was performed with GeneSpring software (Agilent Technologies) on lists of genes altered in p53^(+)^ and p53^(−)^ samples by arsenite treatment. The false discovery rate correction was not applied. This approach identified several genes altered by arsenite in only p53^(+)^ or p53^(−)^ (Table 3). For example, MAP kinase phosphatase 1 (MKP-1) was induced only in p53^(+)^ cells and ubiquitin-conjugating enzyme E2N was induced only in p53^(−)^ cells.

### Arsenite induces cytokinesis failure and apoptosis

An example of a mitotic cell undergoing apoptosis in an arsenite-treated culture is shown in Figure 3B. Mitosis, from the start of prophase to the completion of cytokinesis, normally takes < 30 min in these cells. As can be observed in Figure 3B, 30 min elapsed from the time a cell was in late anaphase/early telophase (panel 1) to when the cell collapsed (panel 8). The metaphase plates holding the chromosomes did not change position while the membrane developed multiple blebs (panels 1–4). The chromosomes appeared to partially decon-dense (panel 5). The cell body collapsed between the plates but the membranes did not fuse to complete cytokinesis (panels 6–8).

## Discussion

Arsenite (as either NaAsO_2_ or As_2_O_3_) is known to activate the G_2_ phase checkpoint (Chen et al. 2002) and to induce mitotic arrest and apoptosis in a variety of cell lines including HeLa (Huang and Lee 1998), U937 (Halicka et al. 2002; McCabe et al. 2000), SV40-transformed human fibroblasts (Halicka et al. 2002; McCabe et al. 2000; States et al. 2002), and several prostate and ovarian carcinoma cells (Uslu et al. 2000). A common feature of the cells in which arsenite induces both mitotic arrest and apoptosis is a functional loss of p53. We have shown that arsenite-induced mitotic arrest occurs in asynchronous cells lacking p53 expression but not in cells expressing p53 (Taylor et al. 2006). In the present study, we have determined whether p53 expression affects the progression of arsenite-treated cells from G_2_ phase into M phase and/or the exit from mitosis.

TR9-7 cells are a well-characterized model to study p53-dependent processes, taking advantage of tetracycline-regulated p53 expression (Adimoolam and Ford 2002; Agarwal et al. 1995; Ford and Hanawalt 1997; Lloyd and Hanawalt 2002; Robles et al. 2001; Taylor et al. 2001) We synchronized these human fibroblasts in G_2_ phase using a multi-step protocol developed by others (Kuhholzer and Prather 2001; Tobey et al. 1990) and adapted to our experiments. The fraction of the cells in G_2_ phase was not as high as some investigators have achieved with normal diploid fibroblasts (Tobey et al. 1990) but higher than that achieved by another group (Kuhholzer and Prather 2001).

Our results indicate that arsenite exposure delays progression from G_2_ phase to M phase in both p53^(−)^ and p53^(+)^ cells. This result is consistent with reported arsenite-induced G_2_ phase delay (McCollum et al. 2005; Park et al. 2001). However, the exit from mitosis was delayed only in p53^(−)^ cells. The mitotic index in arsenite-treated p53^(+)^ cells declined with the same kinetics as in untreated cells. The mitotic index declined more slowly in arsenite-treated p53^(−)^ cells, suggesting that these cells exit mitosis more slowly. The arsenite-treated cells entered apoptosis while futilely attempting cytokinesis. These data indicate that arsenite delays entry into mitosis and progression to anaphase in TR9-7 cells regardless of p53 expression but that exit from mitosis is p53 dependent in arsen-itetreated cells. Thus, both entry into and exit from mitosis is delayed in p53^(−)^ cells. Furthermore, the p53^(−)^ cells undergo apoptosis as a result of failed or abnormal mitotic exit.

As p53 is a transcription factor, we hypothesized that expression profile changes could contribute to delayed mitotic exit kinetics seen in p53^(−)^ cells. To test this, we performed microarray analysis of RNAs prepared from G_2_ phase synchronized p53(+) and p53(^−^) cells harvested at 0-hr and after 3-hr exposure to 5 μM sodium arsenite. *HMOX1* was highly and rapidly induced, independently of p53. *HMOX1* induction is a well-documented response to arsenite (Andrew et al. 2003; Liu et al. 2001). *HMOX1* induction is associated with cellular response to oxidative damage, as the degradation products of heme are known to scavenge free radicals (Abraham 2003).

**Figure 4:**
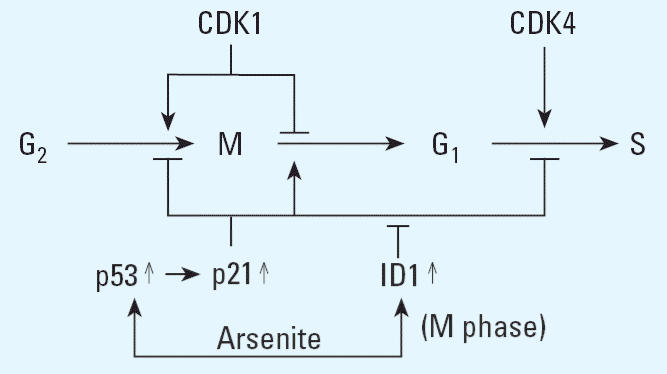
Model for exit from mitotic arrest induced by arsenite and subsequent cell cycle progression dependent on p21^CIP1/WAF1^ and ID1 induction in p53-expressing cells.

The induction of metallothioneins 2A and 3 may also serve a protective role, as metallothionein has been shown to bind arsenicals (Jiang et al. 2003) and metallothionein null mice are hypersensitive to arsenite toxicity (Liu et al. 2000). MKP1, a phosphatase, was induced in p53^(+)^ cells and may serve a protective role as it opposes the activation of SAPK/JNK (Li et al. 2003), a kinase involved in the induction of apoptosis by arsenite (Davison et al. 2004), MKP1 is a transcriptional target of p53 (Li et al. 2003) and has been shown to be arsenite-induced (Li et al. 2001). The expression of ID1 was elevated by arsenite and Western blot analysis showed the extent of induction was greater and more sustained in p53^(+)^ cells. ID1 is a dominant negative inhibitor of transcription that forms inactive heterodimers with basic helix–loop–helix transcription factors. Genes identified as targets of repression by ID1 include those encoding the cyclin-dependent kinase inhibitory proteins *p15^INK4B^, p16^INK4A^,* and *p21^CIP1/WAF1^* (Sikder et al. 2003). Our previous results indicate that the increased sensitivity to mitotic arrest associated apoptosis of p53-deficient cells is due to the lack of p21^CIP1/WAF1^, a transcriptional target of p53 (Taylor et al. 2006). p21^CIP1/WAF1^ may prevent mitotic arrest associated apoptosis in p53^(+)^ cells by allowing for mitotic exit of cells arrested in mitosis. Mitotic exit requires the destruction of cyclin B or inhibition of the cyclin B/CDC2 complex. The promotion of mitotic exit by p21^CIP1/WAF1^ would involve its direct inhibition of cyclin B/CDC2 (Taylor and Stark 2001). However, once p53^(+)^ cells exit mitosis, cell cycle progression would require the inhibition of p21^CIP1/WAF1^. The induction of ID1 by arsenite may serve to repress p21^CIP1/WAF1^ and allow for continued cell cycle progression (Figure 4).

Ectopic overexpression of ID1 immortalizes primary keratinocytes (Alani et al. 1999) and a portion of the ID1 localizes to centrosomes, causing abnormal centrosome number (Hasskarl et al. 2004). Centrosomes serve as the organizational units for the mitotic spindle apparatus. Therefore, an aberrant number of centrosomes may be aneuploidogenic by preventing proper chromosomal segregation. The induction of aneuploidy was posited as the most likely mechanism of arsenic carcinogenesis (NRC 1999, 2001). ID1 also protects against apoptosis through activation of the nuclear factor kappa B signaling pathway (Ling et al. 2003). Higher ID1 levels in p53^(+)^ cells may therefore promote survival. ID1 basal levels are elevated in a variety of cancers, and ID1 is induced during malignant transformation of rat liver cells by arsenic (Chen et al. 2001). Consequently, ID1 induction by arsenite may play an important role in the mechanism of arsenic carcinogenesis by allowing for survival of cells progressing through aberrant mitosis and inducing abnormal chromosome numbers.

Loss of p53 function as seen in many tumors has been suggested to allow cells to bypass cell cycle checkpoints after DNA damage and to enter a nonmitotic state (Erenpreisa and Cragg 2001). Our results suggest p53 plays an important role in prevention of arsenite-induced mitotic arrest and also in apoptosis consequent to the arrest. The prevention of mitotic arrest–associated apoptosis is likely a key event in arsenic-induced carcinogenesis because it predisposes cells to aneuploidy. Understanding the link between p53 and prevention of apoptosis associated with arsenite-induced mitotic arrest will provide essential information regarding the mechanism of arsenic carcinogenesis and also help identify a key target for cancer chemotherapeutics.

## Figures and Tables

**Table 1:** Arsenite-dependent gene induction detected by microarray analysis at 0 and 3 hr after release from G_2_ phase synchrony with FDR applied.^a^

				Fold-change	
UniGene ID	Probe set ID	Gene title	Gene symbol	p53^(+)^	p53^(−)^
517581	33802_at	heme oxygenase (decycling) 1	*HMOX1*	27.2	26.1
504609	36618_g_at	inhibitor of DNA binding 1	*ID1*	6.9	4.3
418241	39081_at	metallothionein 2A	*MT2A*	3.9	3.6
519469	34759_at	solute carrier family 30 (zinc transporter), member 1	*SLC30A1*	3.2	3.1
504609	36619_r_at	inhibitor of DNA binding 1	*ID1*	3.8	2.7
504609	36617_at	inhibitor of DNA binding 1	*ID1*	3.7	2.5
534330	36130_f_at	metallothionein 1E (functional)	*MT1E*	1.9	1.9
513626	31622_f_at	metallothionein 2A	*MT2A*	1.9	1.8
284141	33835_at	TSPY-like 4	*TSPYL4*	1.7	1.9
282326	32168_s_at	Down syndrome critical region gene 1	*DSCR1*	1.6	1.8
37055	1732_at	fibroblast growth factor 5	*FGF5*	1.7	1.6
440939	31623_f_at	metallothionein 2A	*MT2A*	1.6	1.5
105269	33369_at	sterol-C4-methyl oxidase-like	*SC4MOL*	1.4	1.5
467020	1700_at	BCL2 binding component 3	*BBC3*	0.7	0.6
503093	32588_s_at	zinc finger protein 36, C3H type-like 2	*ZFP36L2*	0.4	0.3

a Gene annotations from NetAffx (http://www.affymetrix.com/analysis/index.affx).

**Table 2:** Arsenite-dependent gene induction detected by microarray analysis at 0 and 3 hr after release from G_2_ phase synchrony with FDR not applied. *a*

				Fold-change	
UniGene ID	Probe set ID	Gene title	Gene symbol	p53^(+)^	p53^(−)^
517581	33802_at	heme oxygenase (decycling) 1	*HMOX1*	27.2	26.1
3268	35965_at	heat shock 70k protein 6 (HSP70B’)	*HSPA6*	8.5	6.9
504609	36618_g_at	inhibitor of DNA binding 1	*ID1*	6.9	4.3
520028	1104_s_at	heat shock 70kDa protein 1A	*HSPA1A*	5.8	4.9
418241	39081_at	metallothionein 2A	*MT2A*	3.9	3.6
609521	34759_at	solute carrier family 30 (zinc transporter), member 1	*SLC30A1*	3.2	3.1
504609	36619_r_at	inhibitor of DNA binding 1	*ID1*	3.8	2.7
504609	36617_at	inhibitor of DNA binding 1	*ID1*	3.7	2.5
374950	39120_at	metallothionein 1X	*MT1X*	3.3	4.5
148340	41215_s_at	inhibitor of DNA binding 2	*ID2*	3.1	2.8
799	38037_at	diphtheria toxin receptor	*DTR*	2.8	1.9
153863	1955_s_at	SMAD, mothers against DPP homolog 6	*SMAD6*	2.4	2.0
76884	37043_at	inhibitor of DNA binding 3	*ID3*	2.4	2.5
491611	1137_at	solute carrier family 20 member 2	*SLC20A2*	2.2	2.3
517617	36711_at	v-maf musculoaponeurotic fibrosarcoma oncogene	*MAFF*	2.2	1.9
520819	35303_at	insulin induced gene 1	*INSIG1*	2.1	1.8
196054	37483_at	histone deacetylase 9	*HDAC9*	2.1	1.7

a Gene annotations from NetAffx (http://www.affymetrix.com/analysis/index.affx).

**Table 3:** p53-dependent gene induction by arsenite determined by Venn analysis (FDR not applied).*a*

Unigene ID	Probe set ID	Gene title	Gene symbol	Fold-change
P53^(+)^ cells				
520028	31692_at	heat shock 70kDA protein 1A	*HSPA1A*	4.7
419	34585_at	distal-less homeo box 2	*DLX2*	2.3
171695	1005_at	dual specificity phosphatase 1 (MKP-1)	*DUSP1*	2.1
P53^(−)^ cells				
142912	36799_at	frizzled homolog 2 (Drosophila)	*FZD2*	4.2
50308	40121_at	huntingtin interacting protein 2	*HIP2*	2.5
93002	1651_at	ubiquitin-conjugating enzyme E2C	*UBE2C*	2.2
524630	36604_at	ubiquitin-conjugating enzyme E2N	*UBE2N*	2.2

a Gene annotations from NetAffx (http://www.affymetrix.com/analysis/index.affx).
